# Cholesterol as an Endogenous ERRα Agonist: A New Perspective to Cancer Treatment

**DOI:** 10.3389/fendo.2018.00525

**Published:** 2018-09-11

**Authors:** Ivan Casaburi, Adele Chimento, Arianna De Luca, Marta Nocito, Sara Sculco, Paola Avena, Francesca Trotta, Vittoria Rago, Rosa Sirianni, Vincenzo Pezzi

**Affiliations:** Department of Pharmacy and Health and Nutritional Science, University of Calabria, Cosenza, Italy

**Keywords:** ERRα, cholesterol, cancer metabolism, breast and prostate cancer, colonrectal cancer, adrenocortical carcinoma (ACC), IL-8

## Abstract

The estrogen-related receptors (ERRs) are important members of nuclear receptors which contain three isoforms (α, β, and γ). ERRα is the best-characterized isoform expressed mainly in high-energy demanding tissues where it preferentially works in association with the peroxisome proliferator-activated receptor-γ co-activator 1α (PGC-1α) and PGC-1β. ERRα together with its cofactors modulates cellular metabolism, supports the growth of rapidly dividing cells, directs metabolic programs required for cell differentiation and maintains cellular energy homeostasis in differentiated cells. In cancer cells, the functional association between ERRα and PGC-1s is further influenced by oncogenic signals and induces metabolic programs favoring cell growth and proliferation as well as tumor progression. Recently, cholesterol has been identified as a natural ERRα ligand using a combined biochemical strategy. This new finding highlighted some important physiological aspects related to the use of cholesterol-lowering drugs such as statins and bisphosphonates. Even more meaningful is the link between increased cholesterol levels and certain cancer phenotypes characterized by an overexpressed ERRα such as mammary, prostatic, and colorectal cancers, where the metabolic adaptation affects many cancer processes. Moreover, high-energy demanding cancer-related processes are strictly related to the cross-talk between tumor cells and some key players of tumor microenvironment, such as tumor-associated macrophage that fuels cancer progression. Some evidence suggests that high cholesterol content and ERRα activity favor the inflammatory environment by the production of different cytokines. In this review, starting from the most recent observations on the physiological role of the new signaling activated by the natural ligand of ERRα, we propose a new hypothesis on the suitability to control cholesterol levels as a chance in modulating ERRα activity in those tumors in which its expression and activity are increased.

## General concept

Nuclear receptors (NRs) are a large family of transcription factors that are activated by different signal molecules such as steroids, thyroid hormones, vitamins, retinoic acid, oxysterols, and many other metabolites (1).

A distinct subset of the NRs still remains “orphans” (ONRs) waiting for a defined endogenous ligand. Once activated, NRs together with a multitude of co-factors, drive transcription of genes that control cell proliferation, development, reproduction, and different metabolic phenomena upon which those processes are strictly dependent.

A proper functional control of energy pathways within the cells is supported by the coordination of several transcription factors, including NRs, and associated co-factors. Many members of NR superfamily are involved in these processes since they can activate a specific gene expression network in response to hormonal, nutrient, and metabolite signals coming from distinct physiological (or pathological) conditions (2).

### ERRs structure

The estrogen-related receptors (ERRs) are important members of the ONRs family, deeply involved in the control of energy homeostasis. The ERR subfamily comprises three isoforms, namely ERRα (NR3B1), ERRβ (NR3B2), and ERRγ (NR3B3).

ERRα (45.5kDa, 423a.a.) and β (56.2kDa, 508a.a.) were first identified by screening human male gonad cDNA library with a probe synthesized based on the DNA-binding domain sequence of estrogen receptor alpha (ERα) and ERRα cDNA as a probe, respectively (3). The discovery of the third member, ERRγ (51.3kDa, 45a.a.), was made 10 years later by a different methodological approach (4, 5). In addition, several splice variants of human ERRs have been identified although their physiological role is still to be discovered (6). Despite their name and sequence homology with ERs, they do not bind natural estrogens. Moreover, although the existence of functional crosstalk between ERRs and ERs cannot be ruled out, especially in breast cancer pathology, a deeper investigation revealed that ERRs, particularly ERRα, and ERα have a distinct genomic signature and functions (7).

As members of the nuclear receptor superfamily, ERRs are characterized by a conserved structural and functional organization consisting of:

- A N–terminal region (A/B domain) that contains a ligand-independent transcriptional activation function domain (AF-1). The AF-1 domain of all three ERR isoforms contains conserved motifs that allow the control of the transcriptional activity by post-translational modification such as phosphorylation and sumoylation. In particular, phosphorylation-dependent sumoylation of ERRα within the NH-terminal domain of ERRα and ERRγ negatively affects their transcriptional activity without altering DNA binding with cofactors (8). This mechanism becomes particularly important considering the absence of a specific/natural ligand and the constitutively active conformation of this class of receptors that make their functions dependent on the presence of coactivators and corepressor proteins (see below);
- A central C domain, also known as DNA binding domain or DBD, a sequence shared by almost all three ERR members. DBD allows the recognition of DNA sites through the TNAAGGTCA sequence also known as ERR responsive elements or ERREs. In the DBD there are two digitally shaped helical structures, called “zinc fingers,” in which a zinc ion is coordinated with 4 cysteines. The P-box (proximal box), placed in the first helical structure, enables the recognition of a specific DNA sequence, while the D-box (distal box), placed in the second structure, is involved in the dimerization process on the DNA sequences. Indeed, ERRs can interact with an ERRE sequence as a monomer, homodimer or as a heterodimer consisting of two distinct ERR isoforms (9–11);
- A region linking C to Ligand Binding Domain (LBD);
- A ligand binding domain or LBD, which adopts a transcriptionally active conformation in the absence of any ligand (12);
- A less conserved C-terminal F region, which is present only in some nuclear receptors, including the ERs.

The sequence analysis of all three receptor isoforms reveals a different sequence homology in their domains: all three members show a high sequence identity in their DBD (93–98%); regarding the LBD, ERRα, and ERRβ are less related (57%) while that of ERRβ and ERRγ are closer (73% sequence identity). The latter domain exhibits a 63% sequence identity between ERRα and ERRγ; high level of sequence identity in the A/B domain (60%) characterizes ERRβ and ERRγ (13).

According to their discovery, ERR isoforms are more strongly related to estrogen receptors (ERα and β) than to any other member of the NR superfamily. In particular, the analysis of each individual domain reveals that the DBDs of ERRα and ERα are 70% homologues, but their LBD is only 36% similar, explaining the reasons for the absence of ERRα response to ER ligands (12).

### ERRα: function, regulation, and activity

ERRα discovery determined straightaway questions about its physiological function: ERRα plays a role in embryonic development and its expression level is high in the heart, skeletal muscles, and nervous system. The main physiological role of ERRα is to act as energy sensor to control cellular adaptation to energy demands and to respond to various metabolic stress conditions. Therefore, ERRα is present in high-demand energy tissues, such as muscles and brown adipose tissue. Cells that do not express an activated ERRα cannot produce enough energy at peak demand moments.

In adipose tissue, ERRα promotes the differentiation of mesenchymal stem cells into adipocytes where it regulates energy metabolism. In fact, ERRα increases lipid absorption, β-oxidation, tricarboxylic acid cycle, oxidative phosphorylation, and mitochondrial biogenesis and functions. These metabolic effects are clearly noticeable in all those tissues with high-energy demand including cardiomyocytes and cells of the immune system like macrophages. The prominent role of ERRα in metabolic regulation is underlined by the demonstration that ERRα gene knockout (ERRα-KO) mice have altered fat absorption and metabolism and are resistant to fat-induced obesity (14). Moreover, these mice are not able to adapt to the cold environment and develop cardiac contraction dysfunction. Stress-induced cardiac hypertrophy in ERRα-KO mice is caused by poor ATP synthesis and reduced phosphocreatine deposition (15).

ERRα influences the differentiation of myocytes, T cells, intestinal epithelial cells and osteoblasts. A study showed that ERRα plays a key role in bone development and metabolism during embryogenesis (16). Its mRNA is expressed in murine bone cells during bone formation by endochondral and intramembranous ossification as well as in primary human osteoblasts. ERRα influences the transcription of the gene coding for osteopontin an essential constituent of the mineralized extracellular bone matrix. In the ERRα-KO mice, the loss of ERRα gene expression modestly increased osteoblastic differentiation and spongy bone mineral density, as well as the differentiation of mesenchymal cells into osteoblasts (17).

The constitutive activity of ERRα is structurally related to the presence of a phenylalanine residue (Phe^328^ on helix H3) within its LBD which maintains a conformational arrangement suitable for the interaction with different cofactors (18). ERRα transcriptional activity is dependent on the presence of co-regulatory proteins, which are differentially expressed in various cells and tissues. ERRα preferentially works in association with the peroxisome proliferator-activated receptor-γ receptor (PPARγ) co-activator-1α (PGC-1α) and PGC-1β (18). The functional interaction between ERRα and PGC-1s is highly specific and it is essential for full ERRα receptor activity in most cells. Moreover, ERRα together with PGC-1s integrates many intracellular signals arising from extrinsic/intrinsic metabolic stresses, as well as from growth factors (18).

The activity of ERRα has been examined by the study on the transcriptional co-activators PGC-1α and PGC-1β, which integrate the signals on nutritional and energetic status and drive expression of genes that control mitochondrial biogenesis, oxidative metabolism, and gluconeogenesis.

The ability of the co-activator PGC-1α to favor the activation of target genes is based on its recruitment at the level of gene-specific regulatory sites through physical interactions with NRs and other transcription factors. Regarding the NRs, the amphipathic propellers of PGC-1α bind the hydrophobic region of LBD domain while PGC-1α has a surface that interacts specifically with ERRα. Moreover, ERRα is a powerful transcriptional activator when PGC-1α or PGC-1β are introduced into the system. The tight dependency between ERRα and PGC-1s as the result of the high affinity between ERRα and PGC-1s, suggests that ERRα activity is mainly controlled by the interactions with PGC-1α and β. In addition, PGC-1s are also able to modulate ERRα gene expression (19). Consistent with the ability of PGC-1 co-activators to induce ERRα expression, ERRα mRNA levels are higher in tissues with elevated levels of PGC-1α and β (19).

Given the predominant role of PGC-1s/ERRα transcriptional complex in controlling cellular metabolism, its involvement in the process of tumorigenesis is evident, considering that metabolic adaptations is an hallmark of cancer cells (20). Nevertheless, there are still many aspects to be clarified. In normal cells, the activity of the PGC-1s/ERRα axis is directed to increase cellular metabolism, to support the growth of rapidly dividing cells, to control metabolic programs during cell differentiation and to preserve energy homeostasis once differentiated (13). In cancer cells, the PGC-1s/ERRα complex is a direct target of oncogenic signals that affect metabolic programs in order to favor or attenuate cell growth and proliferation (18). Moreover, PGC-1α-mediated mitochondrial biogenesis and respiration in cancer cells is functionally related to metastatic dissemination (21). Accordingly, PGC-1α gene suppression, by disabling mitochondrial biogenesis and oxidative phosphorylation, decreases the rate of metastasis (21). These findings are also supported by the observations that:

- ERRα together with its coactivator PGC-1α binds to the promoter and regulates the expression of vascular endothelial growth factor (VEGF), a master regulator of tumor invasion and angiogenesis (22, 23);
- ERRα is able to regulate the expression of HIF (hypoxia-inducible factor) in breast cancer cells and to associate with the HIFα/β heterodimer to promote its transcriptional activity on genes involved in angiogenesis and cell migration (23, 24).
- ERRα cooperates with HIF to induce the cancer metabolic reprogramming toward the metastatic-promoting glycolytic state (25).
- ERRα, together with its coactivator PGC-1α, regulates the expression of WNT11, a mechanism involving ERRα in a transcriptional complex with β-catenin (26). In fact, ablation of either ERRα or β-catenin expression decreases the migratory ability of different types of cancer cells. In addition, functional genomic studies have identified further ERRα target genes playing an important role in the process of invasion, migration and tumor vascularization (27).

All these observations highlight the pivotal role of ERRα in many (altered) processes that characterize tumors especially those signaling driving tumor progression and aggressiveness.

### ERRα agonists

The constitutive activity of ERRα does not exclude the existence of a molecule able to modulate its activity enabling the recruitment of cofactors and playing a critical role in the maintenance of energy homeostasis as well as in disease progression. Recently, several synthetic antagonists have been identified (28–30). Moreover, dietary products, including cholesterol, have been reported as potential agonists (31, 32). Suetsugi and collaborators identified agonists through virtual ligand screening on an ERRα ligand binding model based on the crystalline structure of ERRγ-LBD (33). Thus, four ligands, increasing the transcriptional activity of ERRα, have been identified: isoflavones (genistein, daidzein, and biochanin A) and a flavone (trihydroxyflavone) (33). Later, scientists synthesized the potential molecules able to interact with the ligand-domain, guided by ERRα crystalline structure, but they were not able to demonstrate the activity of the agonists (34). Moreover, Peng and collaborators synthesized a series of pyrid (1,2-α) pyrimidin-4 in order to produce more powerful ERRα agonists and to confirm the ability to induce the receptor transcriptional activity (35). These compounds have improved glucose and fatty acid uptake from muscle cells (35) and thus, could have a clinical utility for the treatment of metabolic diseases, including metabolic syndrome and diabetes.

### Cholesterol: the first endogenous ERRα agonist

Recently, an important study investigated the binding ability of ERRα with endogenous lipids (36). To this aim experiments by using chromatography techniques were performed according to previous approaches validated for the study of PPAR with endogenous lipids from the lipidome. The experimental model used is the mouse brain, selected for the high expression of ERRα. The receptor was expressed, purified, and immobilized onto a resin and then incubated with enriching lipidomes. This experimental approach allowed the identification of a single ion that was significantly enriched by the beads bound to ERR-LBD and this ion was identified as cholesterol. Furthermore, to check the specificity of the interaction between ERRα and cholesterol, authors used targeted LC-MS method to increase the detection sensitivity for the lower-abundance sterols (37). The latter results were in agreement with those from lipidomic experiments. Moreover, in order to verify the specificity of ERRα-LBD-cholesterol interaction, a deeper investigation was performed with a competitive binding assay by using diethylstilbestrol (DES), a synthetic ERRα antagonist, that binds to the lipid-binding pocket of ERRα (38). A further confirmation was obtained by the authors with circular dichroism (CD) spectroscopy tests, where cholesterol, DES and the inverse agonist XCT790, all induced a conformational change in ERRα-LBD, while estradiol did not. These results suggested that Cholesterol-ERRα-LBD binding is more than a simple hydrophobic interaction. In addition, dye-labeled cholesterol derivatives were used and, after fluorescence polarization assay, the results showed that cholesterol binds the ligand-binding pocket of ERRα through its hydroxyl group. These findings indicate that cholesterol could exert a functional control of the ERRα activity (36).

### Cholesterol regulates ERRα transcriptional activity

The demonstration that cholesterol impacts ERRα activity comes from the investigation during the osteocalciogenesis process. It has been revealed that ERRα-KO mice have a decreased bone resorption and high bone mass indicating that ERRα is able to promote osteoclast differentiation and activity. The suppression of the osteoclast functions was achieved by the use of statins and nitrogen-containing bisphosphonates, two drugs able to inhibit cholesterol biosynthesis, by blocking the HMG-CoA reductase and farnesyl diphosphate synthase (FPPS), respectively (39, 40). Interestingly, in the absence of ERRα, osteoclast differentiation was neither enhanced by cholesterol nor suppressed by statins. In addition, pharmacological inhibition with XCT790 prevented the effects of cholesterol on parental osteoclasts differentiation. All this data strongly supported the ability of cholesterol to promote osteoclastogenesis acting as an ERRα agonist, whereas statins and bisphosphonates suppressed osteoclastogenesis by reducing cholesterol bioavailability.

Moreover, a further demonstration of the ability of cholesterol to modulate the transcriptional activity of ERRα comes out from conventional transactivation assays by using luciferase as the reporter gene. Indeed, the authors decided to modulate cholesterol abundance in the culture medium. To realize this goal, intracellular sterol levels were reduced by: (i) using lipid-free serum, (ii) adding cholesterol bound to hydroxypropyl cyclodextrin, and (iii) adding the hydrophobic statin, lovastatin.

Cyclodextrin and statin, used as a single agent or as combined treatment, were able to reduce ERRα transactivation. Moreover, the addition of exogenous cholesterol to the samples treated with statins and/or cyclodextrin restored the transcriptional activity of the receptor. This data indicated that cholesterol or a downstream sterol, but not a precursor of the cholesterol pathway, affected ERRα activity. An interesting aspect from the results of the research study by Wei et al. (36), is the need for the presence of cofactors to make ERRα functional, even in the presence of an endogenous ligand. In fact, experiments performed with a silenced PGC-1α gene, revealed that luciferase expression was no longer detectable upon cholesterol treatment even in the presence of ectopic ERRα. These results clearly indicate that cholesterol, and most likely other sterols, affects ERRα function in a PGC-1α-dependent fashion. The authors demonstrated also that ERRα is able to recruit PGC-1α and PGC-1β upon cholesterol addition, but upon cholesterol depletion, PGC-1 coactivators are dissociated from ERRα. Specifically, cholesterol enhances the interaction between ERRα and PGC-1β in osteoclast promoting osteoclastogenesis and bone resorption, while promotes ERRα interaction with PGC-1α in myocytes inducing myogenesis and decreasing statins-induced muscle toxicity.

### Cholesterol-lowering drugs use and outcome of cancer patients

Many experimental evidence strongly suggest that the inhibition of the mevalonate pathway using statin or bisphosphonate drugs has an impact on oncogenic processes such as cell proliferation, tumor progression, and metastatic potential (41). Statins are inhibitors of HMG-CoA reductase, that block the mevalonate pathway progression limiting the accumulation of the final products such as cholesterol, dolichol, isoprenoids, ubiquinone, and isopentenyladenine (42, 43).

Effects of statins on the outcome of patients affected by different types of cancer, including breast (44, 45), prostate (46–48), ovarian (49), lymphoma (50), renal cell carcinoma (51), and colorectal (52, 53) cancer have been examined in the last years. Some of these studies suggests that statins use is associated with longer survival, while others report no benefits. A recent meta-analysis showed that the average effect of statin use is beneficial for overall survival and cancer-specific survival (54). In particular, the study specified that colorectal, prostate, and breast cancers, the three largest cancer-type subgroups, showed a benefit from statins use (54). Interestingly, these three tumors are characterized by high expression of ERRα that, depending on the particular tumor phenotype, is associated with tumor progression and/or worst prognosis (55, 56).

Statins may exert their anticancer effect through several molecular mechanisms: via lowering protein prenylation, reducing tumor cell proliferation and migration, inhibiting rat sarcoma (Ras) signaling, inducing apoptosis through inhibition of Akt phosphorylation and consequently mammalian target of rapamycin (mTOR) down-regulation and other pleiotropic effects on cellular level (57, 58).

Bisphosphonates, especially nitrogen-containing bisphosphonates (N-BPs), are widely used to preserve bone health in patients with cancer thank to their ability to negatively regulate osteoclast-mediated bone resorption that is closely associated with metastasis in different cancer types (59). N-BPs work by interfering with two enzymes belonging to the mevalonate pathway called farnesyl pyrophosphate synthase (FPPS) and geranylgeranyl pyrophosphate synthase (GGPPS). The inhibition of mevalonate pathway by N-BPs results in the accumulation of isopentenyl pyrophosphate (IPP), which is then converted to a cytotoxic ATP analog called ApppI. This last event together with the N-BPs-induced inhibition of protein prenylation causes osteoclast dysfunction and reduced bone resorption. Similarly to studies on statins, epidemiological data projecting bisphosphonates as antitumor agents are still controversial. Although further studies are needed to better elucidate the antitumor effects of bisphosphonates, the general concept that is drawn from current available data (60) is that bisphosphonates reduce cancer metastatic lesions in non-solid as well as in solid tumors. Indeed, in addition to their clinical use to preserve bone tissue, emerging *in vitro* and clinical studies suggest that N-BSs have direct effects on cancer cells including induction of apoptosis, inhibition of proliferation, adhesion, invasion and angiogenesis (60). Synergism with chemotherapeutics and enhancement of immune surveillance expand the pleiotropic effects exerted by N-BPs in oncology.

A new regulator of cholesterol metabolism is represented by protein convertase subtilisin/kexin type 9 (PCSK9) (61) an enzyme produced by hepatocytes and secreted into the plasma to bind to the LDL receptor resulting in lysosomal degradation of the receptor. Consequently, PCSK9 reduces the expression of LDL receptors on the cell membrane thereby decreasing the clearance of LDL-cholesterol. PCSK9 inhibitors (monoclonal-antibodies) have become very useful in statin intolerant patients or when statin therapy is unable to reduce LDL (61).

PCSK9 is mostly expressed in the liver (62), which is one of the most common sites for metastasis, thus the ability of PCSK9 and cholesterol to favor a pro-metastatic environment was investigated. Clinical studies are very poor (63, 64), while the majority of the results comes from preclinical experimental models: the PCSK9-KO mice and melanoma cells (65). In the presence of a reduced expression of PCSK9, the number of hepatic metastases was significantly lower. By contrast, PCSK9-KO and wild-type mice fed with a high cholesterol diet showed an increased number of liver metastasis, suggesting a prominent role of cholesterol in tumor-microenvironment interaction. The results obtained on melanoma cells can be extended to other types of cancer.

The identification of cholesterol as ERRα agonist adds a new anticancer mechanism for statins and nitrogen-containing bisphosphonates that could be further investigated to widen the potential therapeutic alternatives in cancers where ERRα is overexpressed. By contrast, the reduction of LDL-R by PCSK9 inhibitors may result in an increased intracellular content of cholesterol within tumor cells that could sustain ERRα activation. Thus, the use of PCSK9 inhibitors in oncology needs further investigations before the preclinical results can be translated into a clinical setting.

### ERRα, cholesterol, and inflammatory markers

Cholesterol has been shown to inhibit the expression of chemokines, such as CXCL9 and CXCL10, in macrophages (66) while statins and bisphosphonates enhance CXCL9 and CXCL10 gene expression in wild-type (WT) but not in ERRα-KO macrophages. A similar mechanism was observed for other inflammatory markers such as IL-1β and MMP9. These data suggest that chemokine-suppressive effects of cholesterol in macrophages is dependent on ERRα, revealing an anti-inflammatory role for the NR in a cholesterol-rich environment.

However, a deeper analysis should be performed on the role of cholesterol as the modulator of inflammatory markers produced by macrophages. In fact, an interesting paper (67) reported an induction of IL-8 (CXCL8) in response to cholesterol loading in macrophages foam cells, one of the hallmarks of atherosclerosis (68–70). It has been observed that cholesterol loading, in addition to affecting its own uptake, induces several effects in macrophages including the alteration of the cellular metabolism (71, 72), the increase of phospholipids synthesis (73), the increase of apolipoprotein E synthesis and secretion (74), and the enhancement of lipoprotein and apoprotein internalization and degradation (75). All these phenomena contribute to different phases in the progression of atherosclerosis.

In the paper by Wang et al. (67), macrophages were incubated with or without acetylated LDL (acLDL) in the presence or in the absence of an acyl-CoA:cholesterol-acyltransferase (ACAT) inhibitor before evaluating changes in chemokines mRNA content, growth factors, interleukins, and adhesion molecules. Among these genes, a significative increase in mRNA level was observed only for IL-8. Specifically, cholesterol intake by macrophages through scavenger receptor-mediated endocytosis of acLDL enhanced IL-8 expression both at transcriptional and post-transcriptional level.

These observations arouse some deductions concerning the potential positive effect of cholesterol on the expression of the cytokine IL-8, known to be associated with proliferation, angiogenesis, migration, and chemosensitivity of many cancer cells (76). In fact, IL-8 expression has been detected in numerous cancer types, but its value as a cancer biomarker has been poorly investigated, even though it could be relevant for a good number of malignant diseases such as thyroid cancers where IL-8 represents the most deeply investigated chemokine in thyroid tumor microenvironment (77).

For these reasons, we can speculate that cholesterol trough ERRα-dependent signaling regulates production of inflammatory markers in cancer cells as well as in macrophages within tumor microenvironment. Therefore, it seems necessary to delve into this last aspect because ERRα could represent a relevant therapeutic target since this receptor is a key functional factor shared by several oncogenic signals belonging to both tumor cells and microenvironment.

### ERRα, IL-8, and colorectal cancer

Starting from the evidence that ERRα is overexpressed in colorectal (CRC) tumor tissues and cell lines, it was demonstrated that ERRα promotes *in vitro* proliferation and migration (78). Moreover, a close correlation between ERRα protein level and activity and the production of IL-8 in CRC has been found (78). Moreover, chemical inhibition of ERRα activity by treating different CRC cell lines with its inverse agonist, XCT-790, significantly decreased the IL-8 mRNA content, without affecting the expression of other chemokines. These results were further confirmed by ERRα specific silencing experiments. On the other hand, IL-8 expression was upregulated in all CRC cell models characterized by ERRα overexpression.

The same authors, investigating the mechanism by which ERRα regulates the expression of IL-8 in CRC cells revealed that XCT-790 treatment or ERRα gene silencing decreased the promoter activity of IL-8. Moreover, the observation that XCT-790 increased IL-8 mRNA degradation, demonstrated that ERRα regulated IL-8 gene transcription and mRNA stability. Importantly, IL-8 gene silencing suppressed CRC cells proliferation and migration similarly to XCT-790, while a pre-treatment with recombinant IL-8 can rescue the inhibitory effects exerted by XCT-790 on cell proliferation and migration. These results clearly suggest that ERRα activity is directly involved in IL-8-induced CRC cells growth and motility.

Several findings indicate that ERRα activity in CRC could be induced by an excess of cholesterol in the tumor microenvironment. In fact, epidemiologic studies indicate that CRC risk is directly associated with a higher consumption of animal fats and inversely correlated with a diet rich in fruits and vegetables (79, 80). Animal fats diet is also associated with an increased risk of developing chronic intestinal inflammation (81). In fact, an excess of intake in fats of animal origin induces the production of oxidized molecules responsible for lipid oxidation processes able to generate different toxic products for the intestinal epithelial barrier function and the production of pro-inflammatory molecules. All these factors contribute to developing a cancer-prone microenvironment.

The existence of a direct correlation between the levels of cholesterol and the production of IL-8 in the macrophage suggests some interesting hypotheses that could represent the rational basis for further studies: (a) the agonistic action of cholesterol on ERRα in CRC cells could favor the recruitment of co-regulators involved in the enhancement of IL-8 gene expression; (b) a similar mechanism could occur also in macrophages leading the way for new hypotheses on ERRα involvement in the regulation of the inflammatory process within the tumor microenvironment.

### Cholesterol and ERRα in breast, prostate, and adrenocortical cancer

A new potential therapeutic application in a clinical setting controlling cholesterol levels come out from the observations on the role played by ERRα in breast (BC) and prostate (PC) cancers. In BC, high ERRα expression characterizes tumors with poor prognosis (81). Moreover, ERRα mRNA is positively correlated with the oncogene ERBB2 and AIB1 (82) and inversely correlated with that of ERα and progesterone receptor that are good prognostic factors for the anti-hormonal treatment of breast cancer patients. Indeed, depending on the cellular context, ERRα could act promoting or inhibiting transcription (83). Findings suggested that in ER-negative BC, ERRα compensates for the loss of ERα in addition to triggering the expression of ERα-independent genes since it recognizes estrogen response element (ERE) as is the case for vascular endothelial growth factor (VEGF) promoting BC metastasis (23, 24). By contrast, in ER-positive BC cells, ERRα negatively controls ERE transcription by interacting with corepressor such as RIP1. Alternatively, ERRα could promote BC cells growth by enhancing circulating estrogen production. In fact, it has been found that ERRα could activate steroid sulfotransferase (SULT2A1) that works to maintain high level of peripheral dehydroepiandrosteronesulfate (DHEAS), an important metabolite in estrogen synthesis in adrenal tissues. In addition, it has also been evidenced that SULT2A1 inactivates tamoxifen and raloxifene (84). Thus, high ERRα expression in breast cancer by enhancing SULT2A1 activity could also support breast cancer cell resistance to anti-hormonal therapy (84).

The enhanced expression of ERRα has been found also in prostate cancer (PCa) and PCa cell lines (85). A study indicates a positive correlation between ERRα expression and the Gleason score while results from a preclinical study showed that ERRα can promote the hypoxic growth adaptation of prostate cancer cells by interacting with HIF-1α. As above explained, ERRα is also expressed in the bone regulating activity of osteoblasts and osteoclasts, that is implicated into the mixed osteolytic and osteoblastic lesions observed in advanced prostate cancer patients (86).

An increased cholesterol biosynthesis, regulated by sterol regulatory element-binding protein−2 (SREBP-2), is a key player in the initiation and progression of PCa where an enhanced stem cell population was observed (87). Moreover, aberrant cholesteryl ester accumulation in lipid droplets exacerbates cancer invasiveness and characterize high-grade PCa with PTEN loss and consequently, constitutive PI3K/Akt activation promotes metabolic dysregulation where ERRα/PGC-1α, as already mentioned, play a central role (18). In addition, the cholesterol metabolite, 27-hydroxyl-cholesterol (27-OHC) is now recognized as selective estrogen receptor modulator (SERM) which promotes tumorigenesis in ER-positive BC (88). Higher levels of 27-OHC have been reported in ERα-positive breast cancers with respect to normal breast tissue, along with an observed reduction in the 27-OHC metabolizing enzyme such as CYP7B1 (89). Results from *in vivo* experiments demonstrated that 27-OHC alone is sufficient to support estrogenic activity in ER-dependent breast cancer cells (89). Accordingly, an increased growth and metastasis of ER-positive tumors were observed in a mouse model of breast cancer fed only with a cholesterol-rich diet (89). The function of cholesterol as an ERRα agonist may provide the molecular basis and mechanistic insight into clinical studies suggesting that drugs able to lower cholesterol levels (i.e., statins) can be used to treat or prevent breast and prostate cancer.

A different tumor phenotype where cholesterol could have positive growing effects, over its physiological role, is the adrenocortical cancer (ACC). ACC is a very rare and aggressive disease with very limited therapeutic options (90). The pathogenesis of ACC involves the integration of molecular signals and the interplay of different downstream pathways (i.e., IGFII/IGF1R, β-catenin, Wnt, ERα) (91). Our published results indicate that treatment of ACC cell model with XCT-790, to the purpose of reducing ERRα expression, impaired cancer cell growth, both *in vitro* and *in vivo* (92). Our data well correlate with that reporting an increased ERRα expression in ACC compared to normal adrenal and adenoma (93) underling the involvement of this metabolic receptor in ACC biology. Indeed, our unpublished results revealed that treatment of H295R cells with statins caused a significant reduction in cell growth and motility. Although these data are still preliminary, they suggest that cholesterol may affect various biological processes in ACC through the modulation of ERRα activity. Therefore, cholesterol lowering-drug could extend the therapeutic opportunity to fight this rare tumor.

## Conclusion

The ERRα transcriptional activity in normal cells is directed to modulate cellular metabolism, supporting the growth of rapidly dividing cells and to control metabolic programs required for cellular energy homeostasis in differentiated cells and to satisfy energy request during cell differentiation. The recent identification of cholesterol as an endogenous ERRα agonist evidenced that this sterol enhances the interaction between ERRα and PGC-1β in osteoclasts, promoting osteoclastogenesis and bone resorption. Similarly, cholesterol promotes ERRα interaction with PGC-1α in myocytes inducing myogenesis and decreasing muscle toxicity. The discovery of this new molecular mechanism has elucidated the genesis of two important phenomena with an unexplained mechanism: the statin-induced muscle toxicity and the bisphosphonate suppression of bone resorption.

Moreover, the discovery of cholesterol as an agonist of ERRα demonstrated that this receptor works as a metabolic-sensing nuclear receptor distinguishing it from steroid receptors that respond to an acute and steep rise in hormonal levels. Consequently, ERRα is constitutively active because cholesterol is ubiquitous.

This new mechanism calls fresh thinking about the role of ERRα in cancer cells keeping in mind the key role played by this receptor as modulator of cancer metabolism.

It is well known that the metabolic alterations of lipids, carbohydrates, and proteins are one of the hallmarks of cancers (20). In particular, an increase in the glycolytic rate at the expense of oxidative phosphorylation even in the presence of adequate oxygen concentrations (Warburg effect) (94) allows a rapid adaptation of tumor cells to the continuous metabolic changes that, together with the tumor microenvironment, are the driving forces for cancer survival and its evolution. Given the high interconnection between enzymes that regulate the metabolism and the molecular pathways induced by altered oncogenes, research of the key regulators that behave on metabolic adaptations and proliferative, anti-apoptotic, invasive and metastatic responses, could represent elective targets to break down tumors with a single shot. The ERRα could work for this end due to its location at the intersection of dysregulated metabolism and oncogenic pathways.

**Figure 1:**
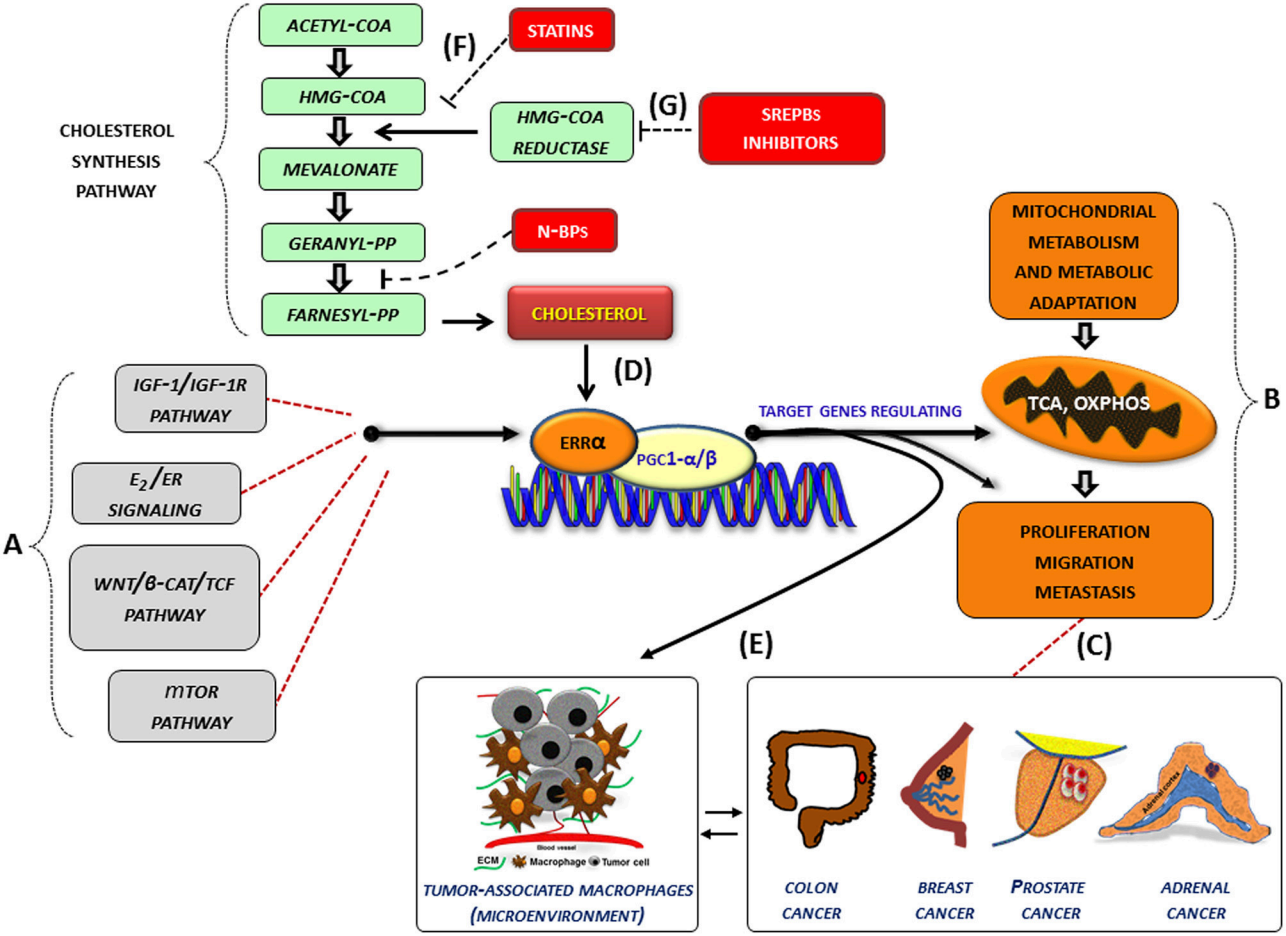
Role of cholesterol as modulator of ERRα action in cancer. **(A–G)** Schematic representation of how cholesterol, as a new ERRα ligand, can contribute to the complex molecular network consisting in the functional cross-talk between oncogenes and oncogenic pathways (IGF-1/IGF-1R, E2/ER, β-catenin/TCF, mTOR) **(A)** that support the overexpression of ERRα. In turn, ERRα, together with its main cofactors (PGC-1α and PGC-1β) and activators, such as cholesterol **(D)**, affects cancer cell metabolism promoting proliferation, migration and, metastasis **(B)** of different tumor phenotypes **(C)**. All these bioenergy-consuming functions are strictly related to **(E)** the cross-talk between tumor cells and some key players of the tumor microenvironment, such as macrophages (tumor-associate macrophages). The use of drugs [statins, **(F)**, N-bisphosphonates, N-BPs, SREBPs inhibitors, **(G)**] able to reduce cholesterol levels and ERRα transcriptional activity could widen the therapeutic opportunities for the treatment of different ERRα overexpressing tumors. More details are explained within the text. E2, estradiol; ER, estrogen receptor; IGF-1/IGF-1R, insulin-like growth factor-1/insulin-like growth factor-1 receptor; WNT, Wingless-type MMTV integration site family member; TCF, T-cell factor; TOR, mammalian target of rapamycin; N-BPs, nitrogen-containing bisphosphonates.

In several cancer cells, the expression and the activity of ERRα, together with its cofactors (PGC-1 α/β), is further influenced by oncogenic signals (IGF1-/IGF1R pathway, estrogen signaling, Wnt/β-cat/TCF, mTOR pathway) (Figure 1A) and can thus be re-directed to induce metabolic programs (Figure 1B) favoring tumor growth and progression. (Figure 1C). In this context, an increased level of cholesterol, through the new molecular mechanism, supports all tumor-related processes. (Figure 1D). Accordingly, high levels of cholesterol are associated with an increased risk of different type of cancers including breast, prostate (95) and CRC (96).

Although epidemiological data on the correlation between cholesterol and cancer are conflicting, the preclinical results positively highlight different molecular aspects revealing how oncogenic growth signaling meet the bioenergetics and biosynthetic demands of rapidly proliferating tumor cells. In fact, altered cholesterol pathway in cancer could be reached through different mechanisms. One of the most important is the constitutive activity of the oncogenic PI3K/AKT/mTOR signaling pathway that enhances intracellular cholesterol levels by: (i) inducing cholesterol synthesis through the activation of the transcription factor SREBP (sterol regulatory element binding proteins); (ii) inducing LDL receptor–mediated cholesterol import; (iii) inhibiting ABCA1-mediated cholesterol export. Moreover, high-energy demanding cancer related process are strictly related to the cross-talk between tumor cells and some key players of the tumor microenvironment (TME), such as macrophages (TAM, tumor-associated macrophage), that in turn, fuels cancer progression through the formation of an inflammatory milieu characterized by the production of different cytokines such as IL-1, IL-6, and IL-8 among others. The latter, as above reported, could be a target of ERRα action (Figure 1E). For most solid tumors, infiltration by inflammatory cells such as macrophages is associated with poor prognosis (97, 98).

The links between inflammation and cholesterol are best exemplified by atherosclerosis, but similar mechanisms may also contribute to other metabolic disorders including cancer. It is noteworthy that cholesterol accumulation in TAM triggers the phenotype switch from M1, antitumorigenic, to M2-like macrophage, protumorigenic (99, 100).

Based on these considerations, the use of therapeutic strategy aimed to reduce cholesterol levels, such as statins (Figure 1F) or drugs targeting the SREBP metabolic pathways (Figure 1G), could be a promising option to counteract metabolic rewiring in cancer cells where ERRα plays a pivotal role.

In conclusion, identification of cholesterol as an endogenous ERRα agonist has already elucidated the most likely mechanisms underlying the side-effects induced by statins and bisphosphonate, but at the same time, it gives new perspectives to be further investigated in order to explore new therapeutic options for the treatment of ERRα overexpressing tumors. This alternative approach could bring additional benefits to the treatment of tumors that have already adopted successful therapies, but especially for those tumors, such as ACC, which are characterized by a limited or failed therapeutic choice.

## Author contributions

IC, AC: literature revision and drafting of the article. ADL, MN, SS, PA, FT, and VR: drafting of the article. RS and VP: critical revision of the article and final approval.

### Conflict of interest statement

The authors declare that the research was conducted in the absence of any commercial or financial relationships that could be construed as a potential conflict of interest.
